# Gender Differences in Sleep and Mental Health among Saudi Adolescents

**DOI:** 10.1155/2021/5513817

**Published:** 2021-09-10

**Authors:** Ahmad Mamoun Rajab, Tawfik Mamoun Rajab, Amjad Chamsi Basha, Abdullah Murhaf Al-Khani, Mohamed Abdelghafour Ali, Saed Enabi, Mohamed Saddik Zaghloul, Abdulrahman Almazrou, Juliann Saquib, Nazmus Saquib

**Affiliations:** College of Medicine, Sulaiman Al Rajhi University, Al Bukairyah, 51941, Saudi Arabia

## Abstract

Among adolescents, mental health issues (i.e., stress and depressive symptoms) negatively affect sleep. We assessed whether the association between mental health and sleep varied between genders among Saudi adolescents. A total of 2206 school students (grades 7-12) from 40 randomly selected schools in four cities of Al-Qassim province in Saudi Arabia participated in this cross-sectional study. The survey assessed demography, lifestyle, sleep (12-item Medical Outcomes Study Sleep Scale), depression (Depression, Anxiety, Stress Scale (DASS-21)) and stress (10-item Perceived Stress Scale). Adjusted associations with sleep were tested with linear and logistic regressions. Of the sample, 55% were girls, and their average sleep score was lower than that of the boys (58.7 vs. 63.4, *p* < 0.001). Girls had worse mental health than boys; the proportion of girls with both severe stress and severe depressive symptoms was three times higher than that of the boys (12% vs. 4%, *p* < 0.001). For both boys and girls, those with severe depressive symptoms only or both severe depressive symptoms and severe stress had significantly lower sleep scores than those who had neither of the two conditions (reference group). On the other hand, among those who had severe stress only, the sleep score was significantly lower for the girls (*p* = 0.002) than for the boys (*p* = 0.19). Overall, girls had a significantly lower sleep score and worse mental health than boys. The association between mental health and sleep significantly differed between the sexes. Severe stress was negatively associated with sleep in girls but not in boys.

## 1. Introduction

Sleep plays a crucial role in the growth and development of adolescents, including their learning and cognition [1]. They may not be getting adequate sleep in this age of information technology due to an overreliance on electronic gadgets. A recent national survey showed that among Canadian children and adolescents, one in three sleeps less than is recommended for their age [2]. Around two-thirds of Saudi adolescents are sleep deprived and suffer from sleep disturbances [3]. Inadequate (or poor quality) sleep will likely put them at higher risk of psychological distress/disorders [4]. It also affects adolescents in a myriad of other negative ways, such as inattentiveness, reduced academic performance, delinquent behavior, accidents, injury, obesity, and metabolic disturbances, to name a few [4, 5].

There is evidence of a gender difference in sleep and mental health among adolescents. Girls are more likely to develop depression than boys; this association becomes apparent in adolescence but persists throughout adulthood [6]. Similarly, girls are more likely than boys to report sleep difficulties, such as insomnia, restless leg syndrome, and sleep dissatisfaction [7, 8]. A few mechanisms have been proposed to explain this gender difference. For example, girls go through unique pubertal changes, such as menstruation, which negatively affect sleep duration [9]. Sex hormone fluctuations (estrogen and progesterone) impact sleep; in particular, the steep rise in progesterone levels during the last week of the menstrual cycle is postulated to cause sleep fragmentation [10, 11]. Lack of sleep, in turn, activates the hypothalamic-pituitary-adrenal stress pathway, to which girls are particularly sensitive [12]. Moreover, girls respond differently to various environmental, social, and emotional stimuli than boys [13]. Their lifestyle (e.g., activity, diet, and screen time) is also different from boys, which may further add to the gender difference in sleep and mental health [14].

Saudi Arabia offers a unique environment to study gender differences in sleep and mental health among adolescents. Saudi Arabia's demography is heavily tilted towards the young; adolescents comprise 15% of the total population [15]. Residents are more likely to be physically and socially active in the nighttime because of hot weather in the daytime. This reduces the sleep duration for adolescents who have school in the morning. Due to the conservative nature of the society, there are not as many outdoor activities (e.g., open grounds for sports and group gatherings) available for girls as there are for boys. Such activities are considered helpful for coping with stress and depression [16].

Although several studies have reported significant sleep problems among Saudi adolescents, hardly any studies have assessed the relationship of sleep with mental health, and no study has evaluated whether that relationship might differ by gender. For example, Merdad et al. reported that as many as 66% and 37% of Saudi adolescents might be suffering from sleep disturbance and excessive daytime sleepiness, respectively [3]. Other available studies also reported a general trend of poor sleep among Saudi adolescents [3, 17–20].

This study randomly drew a large sample of school-going adolescents from the central region of Saudi Arabia and assessed sleep and mental health with validated tools. The study objectives were to determine (1) their mental health profile according to stress and depression, (2) their sleep quality and pattern, (3) the relationship between their sleep and mental health, and (4) whether that relationship varied by gender. One hypothesis was that adolescents who suffer from severe stress or depression are more likely to be poor sleepers than those who do not have either of the two conditions.

## 2. Materials and Methods

### 2.1. Participants and Data Collection

The methodology (eligibility criteria, sample size, and sampling procedure) was described in detail in a previous publication [21]. Briefly, 2675 students who were enrolled in governmental schools (grades 7-12) in four main cities of the central region of Saudi Arabia participated in this 2018 survey. These students attended a total of 40 gender-segregated schools selected randomly from the list of all schools (*n* = 190) in these cities. Research assistants visited the classrooms, explained the study purpose to the students, invited them to the study, obtained informed consent, and administered the paper and pencil survey. The protocol was reviewed and approved by the ethics committee of the regional directorate of the Ministry of Education.

### 2.2. Exposures

The validated Arabic version of the Perceived Stress Scale (PSS) (Cronbach's alpha coefficient 86.4) was used to assess stress [22, 23]. It has ten items rated on a 5-point Likert scale, ranging from 0 (never) to 4 (very often). The items consist of both positive and negative factors; the scores of the negative items were reversed and recoded during analysis. Total scores ranged from 0 to 40, with higher scores indicating higher levels of stress. Participants were categorized as having low (≤13), moderate (14-26), or severe (high) stress (≥27) [24].

The validated Arabic version of the Depression, Anxiety, Stress Scale (DASS-21) questionnaire (Cronbach alpha 0.81) was used to assess depression [25, 26]. Each of the seven items for depression was rated on a 4-point scale ranging from 0 (never) to 3 (almost always). The summary score for each participant was multiplied by 2, and established cut-off values were used to categorize the participants into normal (0–9), mild (10–13), moderate (14–20), and severe (≥21) depression.

The 3-level perceived stress (i.e., low, moderate, and severe) and the 4-level depression (i.e., normal, mild, moderate, and severe) variables were both made binary by using severe as the cut-off point and collapsing all the levels below severe into one. This allowed the creation of a “mental health profile” variable wherein a participant had only severe stress, only severe depression, both severe stress and depression, or neither severe stress nor depression.

### 2.3. Outcome

Sleep was assessed with the Medical Outcomes Study Sleep Scale (MOS-SS) [27]. It has 12 items that measure key sleep parameters across six domains: sleep disturbance (4 items), sleep adequacy (2 items), somnolence (3 items), snoring (1 item), awakening due to shortness of breath or with headache (1 item), and quantity of sleep (1 item) [28]. Nine items from sleep disturbance, sleep adequacy, somnolence, and awakening domains were used to calculate the Sleep Problems Index (SPI) II, which reflects a person's overall sleep quality. SPI was analyzed both as a continuous variable and as a binary variable (above median = good sleepers, below median = poor sleepers). The final scores were interpreted as in the User's Manual for the Medical Outcomes Study (MOS); higher scores in any domain indicate better sleep and more favorable outcomes [29]. The MOS-SS has demonstrated positive psychometric properties in a broad range of populations [27, 28].

### 2.4. Covariables

A set of variables was selected from the literature review for analysis: age, gender, parents' marital status (married, divorced/widowed), academic grade (excellent, very good, good, pass, fail), current smoking status (nonsmoker, smoker), self-reported diet (very healthy, somewhat healthy, somewhat unhealthy, very unhealthy), self-reported physical activity (very active, somewhat active, somewhat inactive, very inactive), and screen time per day (>3 hours, 2-3 hours, 1-2 hours, <1 hour).

### 2.5. Statistical Analysis

The data was analyzed in SPSS (version 25), and all tests were two-tailed with an alpha of 0.05. Out of the sample of 2675, only 2206 were used in the analysis as the records of 23 individuals were mostly empty, and the records of another 446 individuals (52.5% boys and 47.5% girls) had missing data on sleep and mental health. Total sleep score, summary stress score, and summary depression score were graphed separately for boys and girls for a visual comparison of distribution. Boys and girls were compared and contrasted (i.e., chi-square or *t*-test depending on the nature of variable) in relation to demography and other characteristics. The proportion with mental health conditions (i.e., severe stress or severe depression alone, both, or none), average total sleep score, and average domain-specific sleep score were calculated and graphed for the entire sample and by gender. The average total sleep score and average domain-specific sleep score were calculated and compared across the categories of mental health conditions (i.e., severe stress only, severe depression only, both severe stress and depression, none) with analysis of variance (ANOVA).

We planned for a multilevel regression to determine the association between mental health and sleep due to the anticipated hierarchical nature of the data (i.e., respondents were nested within class, class within school, and school within city) [21]. However, analysis showed little or no clustering effect (city and grade explained <1% of total variance). Consequently, we opted for a traditional regression approach (i.e., multiple regression). Prior to model building, a formal test of interaction between mental health and gender was conducted with sleep as the outcome. Models were presented separately for boys and girls because of the significant interaction.

The multiple linear regression identified the correlates of continuous sleep score, SPI; the slope estimate (beta) with its associated *t*- and *p* values for each variable was reported. The multiple logistic regression identified correlates of binary sleep (good vs. poor sleeper); odds ratios and their corresponding 95% confidence intervals were reported for each variable.

A set of covariables, chosen a priori, were used for model building (i.e., age, level, city, body mass index, mental disturbance, academic performance, physical activity, diet, smoking, family structure, and screen time). Of these variables, those that attained a *p* value of <0.25 in the univariate model were selected for the adjusted models; the chosen variables were mental disturbance, academic performance, physical activity, diet, smoking, family structure, and screen time.

## 3. Results

### 3.1. Sample Characteristics

The analytic sample consisted of 2206 adolescents (boys: 45%, girls: 55%); their mean (SD) age was 16.01 years (standard deviation 1.59). Around 10% were from a single-parent home (i.e., divorced parents or one parent passed away), 4% were smokers, 46% reported consuming an unhealthy diet, and 20% reported being physically inactive. The mean (SD) of their body mass index (BMI) was 22.3 (5.2). Two-thirds (63%) reported >3 hours of screen time per day. Nearly all (98%) reported having a good grade, with 51% reporting an excellent grade. The mean (SD) total sleep score (MOS-SUM) was 60.8 (17.14); 49% were poor sleepers (sleep score below median) (Table 1). The distribution for total sleep, summary stress, and summary depression scores was comparable between boys and girls (Supplementary Figure 1).

### 3.2. Gender Differences in Covariables

Girls were, on average, older, had lower BMI and better academic performance (excellent grade: 54% vs. 48%), and were less likely to be smokers (0.8% vs. 7%) than boys. However, they were more likely to come from a single-parent home than from an intact family (12% vs. 8%). Their diet was reported to be unhealthier (47% vs. 43%), and they spent more time in front of screens as compared to boys (65% vs. 60%, >3 hours) (Table 2).

### 3.3. Exposures

**Figure 1:**
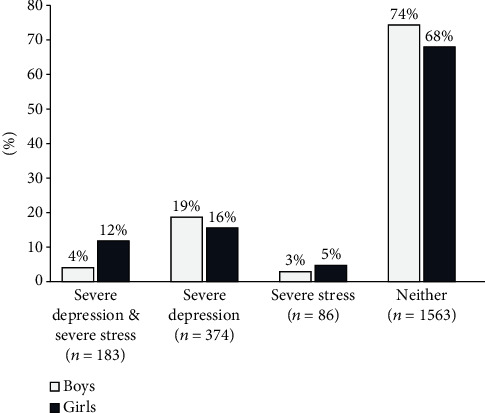
Mental health profile of the students (severe depression and stress, severe depression only, severe stress only, and no severe depression or stress) stratified by gender.

Around three-fourths (74%) of the participants were experiencing stress (moderate = 61.9%, high = 12.1%), and 60% suffered from some degree of depressive symptoms (mild to moderate = 34%, severe to extremely severe depression = 25%). Approximately 17% had only severe depressive symptoms, 4% had only severe stress, and 8% had both severe depressive symptoms and severe stress (Figure 1).

### 3.4. Gender Differences in Exposures and Outcome

The proportion of participants with severe stress or depressive symptoms was higher among girls than boys (Table 2). In addition, thrice as many girls had both severe stress and severe depressive symptoms as boys (12% vs. 4%, *p* < 0.001). It was more common for girls to have severe stress alone (5% vs. 3%, *p* < 0.001), but less common to have severe depressive symptoms alone (16% vs. 19%, *p* < 0.001) as compared to boys (Figure 1).

**Figure 2:**
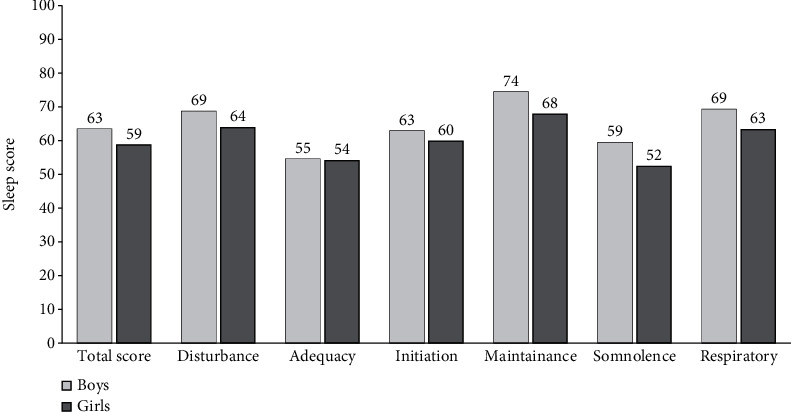
Sleep domain mean scores stratified by gender (*n* = 2206).

The mean sleep score for girls was significantly lower than the corresponding score for boys (58.7 vs. 63.4, *p* < 0.001); the mean scores for all individual sleep domains were also lower for girls than boys (sleep disturbance, initiation, adequacy, maintenance, somnolence, and respiratory problems). The differences were significant in all domains (*p* < 0.001) except for sleep adequacy (*p* = 0.72) (Figure 2).

### 3.5. Sleep Score by Mental Health

The mean sleep score was significantly lower for adolescents who had both severe stress and severe depressive symptoms (45.22, *p* < 0.001) than for adolescents who had neither of those two conditions (reference group: 65.25). Compared to the reference group, the mean sleep score was also lower for those who had severe stress alone (57.6, *p* < 0.001) or who had severe depressive symptoms alone (50.78, *p* < 0.001). The reference group had significantly better sleep scores in all sleep-related domains (data not shown).

### 3.6. Gender, Mental Health, and Sleep

Among the boys, those with severe depressive symptoms only or with both severe depressive symptoms and severe stress had significantly lower sleep scores (-15.3 and -18.3, respectively) than the reference group. The corresponding results were similar for the girls. The gender difference was observed among those who had severe stress only: the sleep score was not significantly lower for boys (*p* = 0.19), but it was significantly lower for the girls (*p* = 0.002) (Table 3).

Among boys, those with severe depressive symptoms only or with both severe depressive symptoms and severe stress were significantly more likely (6.1 and 5.8 times, respectively) to have a sleep score below the median than those who had neither of the two conditions (reference group). The corresponding results were similar for the girls, but the magnitude was lower. The difference was that among those who had severe stress only, the likelihood to have a sleep score below the median was not significant for boys (*p* = 0.08), but it was significant for girls (*p* = 0.004) (Table 4).

## 4. Discussion

This study assessed mental health and sleep quality with a large sample of adolescents (grades 7-12) from Saudi Arabia and tested whether the association between these two variables varied by gender. The salient findings were that (1) 12% of the sample had severe stress alone, 34% had severe depressive symptoms alone, and 8% had both severe stress and severe depressive symptoms; (2) 48.5% of the sample were poor sleepers; (3) girls, overall, had a poorer mental health profile and lower sleep scores in all domains compared to boys; and (4) the association between mental health and sleep significantly varied between the sexes, i.e., severe stress alone made the girls more likely than the boys to be poor sleepers, whereas severe depression alone made the boys more likely than the girls to be poor sleepers.

This study adds to the evidence that a large portion of Saudi adolescents may be suffering from severe stress, particularly girls [30, 31]. A recently published study among expatriate adolescents from Al-Qassim, Saudi Arabia, reported a high prevalence of psychological distress (54%), indicating that non-Saudi adolescents may not be immune from severe stress either [32]. This high prevalence of severe stress likely stems from various physiological and psychological changes that adolescents go through during this stage of life [32]. Some variation of the estimate may also originate from the difference in tools used in the studies and differences in sample composition.

The prevalence of depressive symptoms among adolescents reported in previous studies ranged from 30% to 45% [30, 33, 34]. The prevalence of depressive symptoms in the current study is much higher (overall = 59%, boys = 54.3%, and girls = 63%). This discrepancy may have resulted from the various assessment tools and/or cut-off values for depressive symptoms used in the studies. Additionally, geographical location may also have played a role in the observed difference in depression as none of these studies were conducted in Al-Qassim. The current study was conducted in Al-Qassim, a rich region in Saudi Arabia; a generally higher economic status among the populace may have rendered the boys and girls more sensitive to questions about thoughts and feelings.

The poor sleep quality reported in our study supports the existing Saudi literature on sleep [17–20]. Nasim et al. [17] found that around half of Saudi adolescents (46%) are sleep-deprived (<7 hours of sleep/day); they found that girls were more likely to be sleep-deprived than boys (OR 1.23, CI 1.14-1.34). Population-based studies in the United States have shown that adolescent girls report poorer sleep and more insomnia compared to boys [35, 36]. One explanation might be that girls tend to undergo pubertal changes earlier than boys. These changes are associated with a shift in the circadian rhythm—the body's natural cycle that regulates the timing of sleepiness and wakefulness—resulting in sleep phase delay [37]. This may account for the gender difference in sleep quality seen in the current study.

Several studies assessed whether the association between mental health and sleep differed between genders. More often than not, the studies found that there is indeed a gender difference. Girls are more likely than boys to show poor outcomes in sleep and mental health [38, 39]. For example, Schneiderman et al. [39] reported that adolescent girls had worse sleep and mental health than adolescent boys. The current study reports an additional finding: severe stress affects sleep significantly in girls but not in boys. This finding may not be surprising given that girls sleep longer and report higher levels of perceived stress compared to boys [40]. For example, in a study among urban adolescents, greater cortisol increments and stronger effects of sleep problems on cortisol reactivity were detected among girls than boys [41]. Similarly, for girls, greater cortisol reactivity is associated with more anxiety and depressive symptoms [42].

The present study had several strengths, including a large and gender-representative sample as well as a high response rate to the survey. An additional strength was the use of a comprehensive and validated sleep scale.

### 4.1. Limitations

The results of this report should be interpreted with the following caveats: (1) no direct causality between mental health and sleep can be drawn because of the cross-sectional study design; (2) the sample did not include students from private or international schools; (3) the assessment of stress, depression, and sleep parameters was self-reported and therefore subjective in nature; and (4) sleep information was likely subject to recall bias because participants had to evaluate their sleep during the prior month.

## 5. Conclusions

There was a significant association between mental health and sleep quality among Saudi adolescents, and severe stress affected sleep significantly more among girls than boys. The study implications are that school authorities could screen adolescent students, especially girls, to identify those who have severe stress and/or depressive symptoms and provide them with the needed counseling or refer them for further medical attention. Additionally, school authorities could conduct a health campaign to promote sleep and to educate the students on the importance of good sleep habits.

## Figures and Tables

**Table 1:** Baseline information of the study sample (*N* = 2206).

Variables	Frequency (%) or mean ± SD
Age (years)	16.01 ± 1.59
Gender	
Boys	991 (44.9)
Girls	1215 (55.1)
Level	
Intermediate school	754 (34.2)
High school	1452 (65.8)
Grade	
Excellent	1130 (51.2)
Good/very good	1031 (46.7)
Pass	37 (1.7)
Fail	8 (0.4)
Single parent (divorced/widowed)	227 (10.3)
Smoker	78 (3.5)
Unhealthy diet	1005 (45.6)
Physical inactivity	435 (19.7)
Screen time (hours)	
>3	1392 (63.1)
2-3	346 (15.7)
1-2	297 (13.5)
<1	171 (7.8)
Stress	
High	267 (12.1)
Moderate	1366 (61.9)
Mild	573 (26)
Depression	
Severe/extremely severe	557 (25.2)
Mild/moderate	747 (33.9)
Normal	902 (40.9)
Total sleep score	60.8 (17.14)
MOS-SUM	
Poor (below median)	1071 (48.5)
Fair (above median)	1135 (51.5)

MOS: Medical Outcomes Study.

**Table 2:** Sample characteristics across gender (*n* = 2206).

		Gender				
		Boys		Girls		
		Mean	SD	Mean	SD	*p*
Age		15.9	1.5	16.2	1.6	<0.001
BMI		22.84	5.73	22.03	4.75	<0.001
Sleep Problems Index II		63.42	16.92	58.73	17.05	<0.001

		Count	%	Count	%	*p*

Level	Intermediate	408	41.2%	346	28.5%	<0.001
	High school	583	58.8%	869	71.5%	
Grade	Nonexcellent	513	51.8%	563	46.3%	0.011
	Excellent	478	48.2%	652	53.7%	
Family structure	One parent	82	8.3%	145	11.9%	0.005
	Both parents	909	91.7%	1070	88.1%	
Physical activity	Very inactive	45	4.5%	59	4.9%	<0.001
	Somewhat inactive	146	14.7%	185	15.2%	
	Somewhat active	517	52.2%	747	61.5%	
	Very active	283	28.6%	224	18.4%	
Diet	Very unhealthy	98	9.9%	146	12.0%	<0.001
	Somewhat unhealthy	331	33.4%	430	35.4%	
	Somewhat healthy	443	44.7%	568	46.7%	
	Very healthy	119	12.0%	71	5.8%	
Smoking	Smoker	68	6.9%	10	0.8%	<0.001
	Nonsmoker	923	93.1%	1205	99.2%	
Screen time	>3 hours	597	60.2%	795	65.4%	0.006
	2-3 hours	180	18.2%	166	13.7%	
	1-2 hours	127	12.8%	170	14.0%	
	<1 hour	87	8.8%	84	6.9%	
Perceived stress	Low stress	311	31.4%	261	21.5%	<0.001
	Moderate stress	611	61.7%	754	62.1%	
	High stress	69	7.0%	200	16.5%	
Depression	Normal	453	45.7%	449	37.0%	<0.001
	Mild to moderate	313	31.6%	434	35.7%	
	Severe to extremely severe	225	22.7%	332	27.3%	
Sleep Problems Index II	Poor sleepers	417	42.1%	654	53.8%	<0.001
	Good sleepers	574	57.9%	561	46.2%	

**Table 3:** Multivariate linear regression with total sleep score as outcome.

Mental disturbance	Boys			Girls		
	Beta	*t*	*p*	Beta	*t*	*p*
S Dep+S stress	-18.351	-11.089	<0.001^∗^	-16.194	-8.869	<0.001^∗^
S Dep only	-15.011	-11.960	<0.001^∗^	-11.371	-9.002	<0.001^∗^
S stress only	-5.759	-2.347	0.19	-7.361	-3.104	0.002^∗^
No Dep+no stress	Ref			Ref		

S: severe; Dep: depression; ∗ denotes significance. The model was adjusted for the following: physical activity, diet, smoking, family structure, grades, and screen time.

**Table 4:** Multivariate logistic regression with poor sleep (below median total sleep score) as outcome.

Mental disturbance	Boys			Girls		
	OR	95% CI	*p*	OR	95% CI	*p*
S Dep+S stress	5.786	3.375–9.918	<0.001^∗^	5.263	2.954–9.378	<0.001^∗^
S Dep only	6.074	4.106–8.984	<0.001^∗^	3.545	2.488–5.052	<0.001^∗^
S stress only	1.822	0.942–3.523	0.075	2.577	1.360–4.882	0.004^∗^
No Dep+no stress	Ref			Ref		

S: severe; Dep: depression; ∗ denotes significance. The model was adjusted for the following: physical activity, diet, smoking, family structure, grades, and screen time.

## Data Availability

The data used to support the findings of this study are available from the corresponding author upon request.

## Supplementary Materials

Supplementary Materials Supplementary Figure 1: histogram of total sleep score, summary stress, and summary depression score for boys and girls.
