# Supplementary material for: Gender Differences in Sleep and Mental Health among Saudi Adolescents
Source: Sleep Disord. 2021 Sep 10;2021:5513817. doi: 10.1155/2021/5513817 (PMC8448587; doi:10.1155/2021/5513817)

Supplementary Figure 1: Histogram of total sleep score, summary stress, and summary depression score for boys and girls


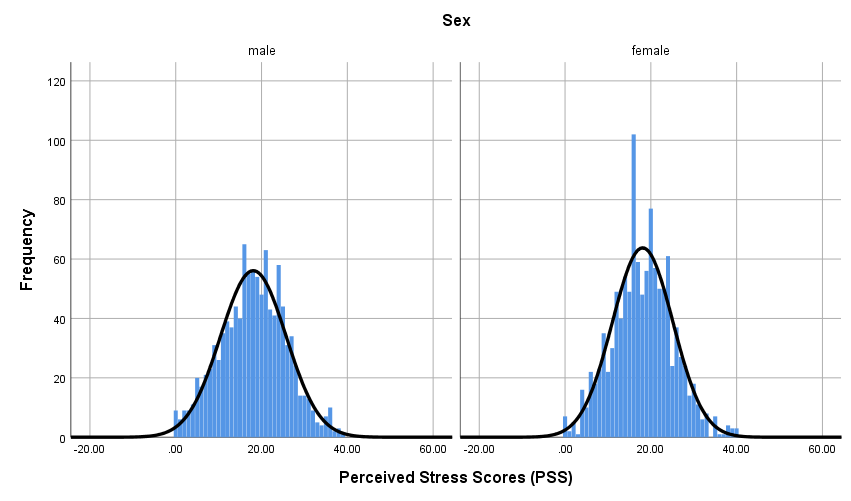


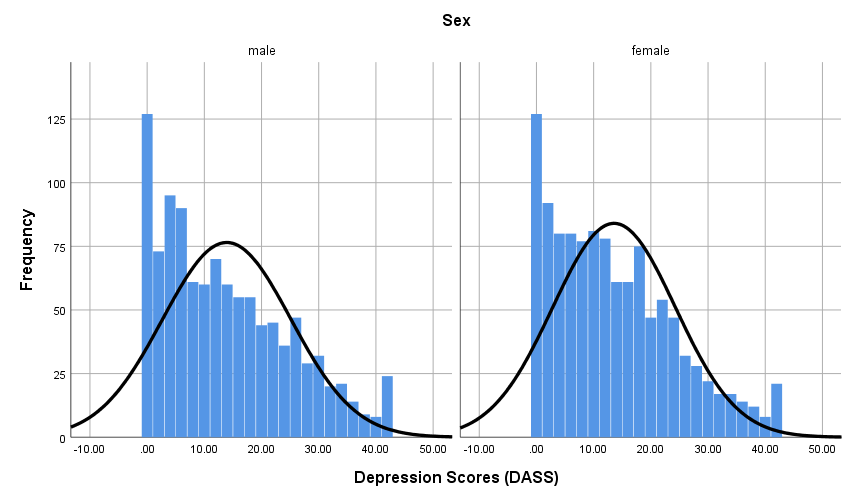


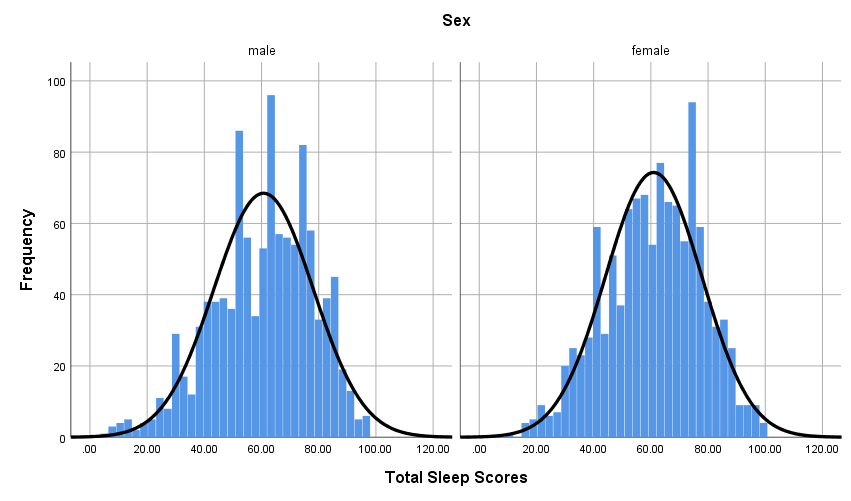


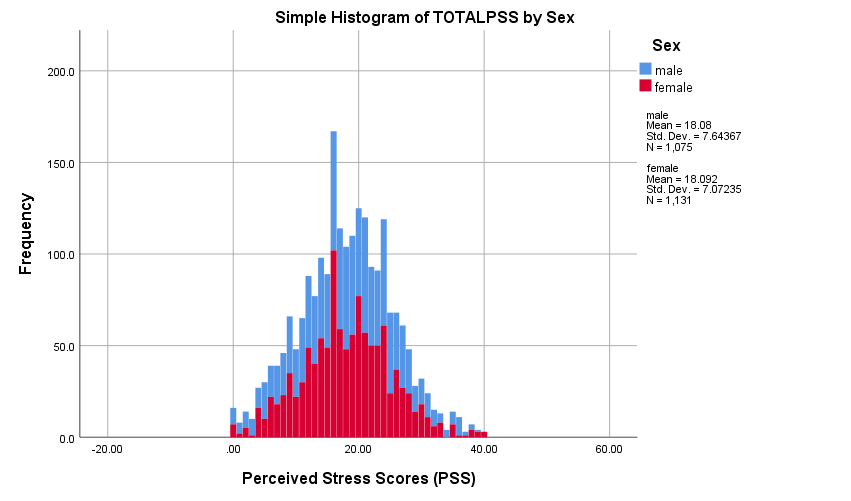


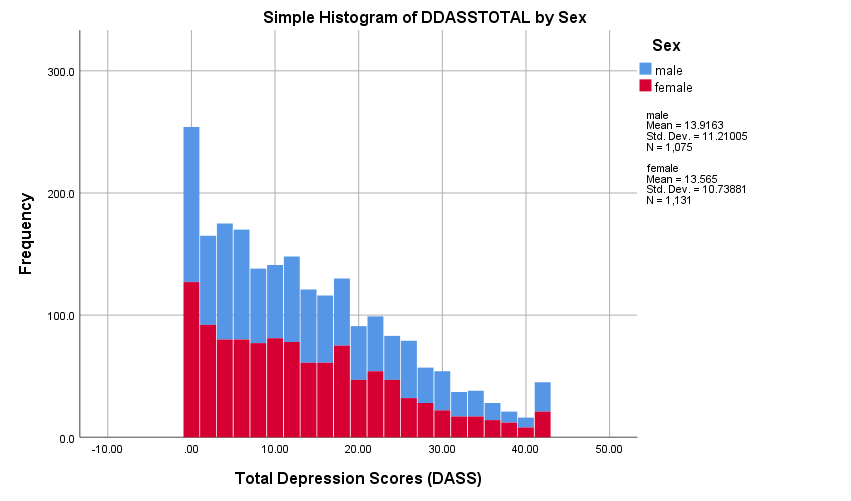


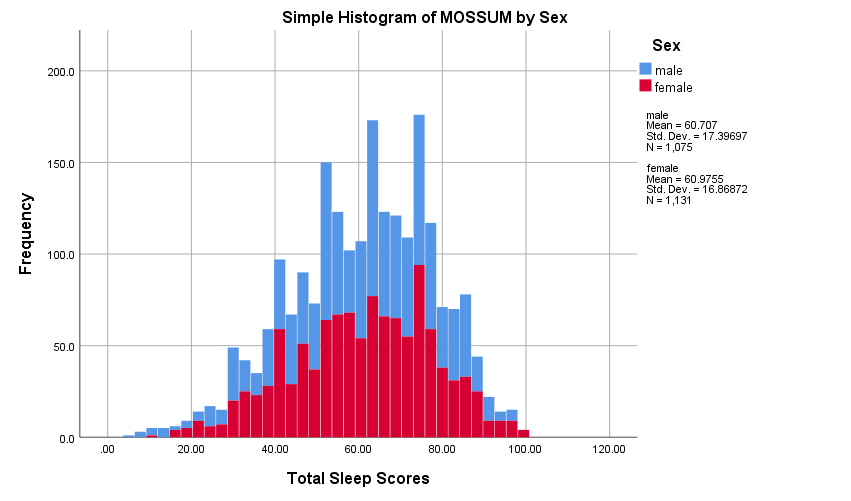

Supplement: Supplementary Materials — Supplementary Figure 1: histogram of total sleep score, summary stress, and summary depression score for boys and girls. [file 5513817.f1.docx]
